# Confident difference criterion: a new Bayesian differentially expressed gene selection algorithm with applications

**DOI:** 10.1186/s12859-015-0664-3

**Published:** 2015-08-07

**Authors:** Fang Yu, Ming-Hui Chen, Lynn Kuo, Heather Talbott, John S. Davis

**Affiliations:** 1 Department of Biostatistics, University of Nebraska Medical Center, Omaha, 68198-4350 NE USA; 2 Department of Statistics, University of Connecticut, Storrs, 06269-4120 CT USA; 3 Department of Biochemistry and Molecular Biology and Department of Obstetrics and Gynecology, University of Nebraska Medical Center, Omaha, 68198-5870 NE USA; 4 VA Nebraska-Western Iowa Health Care System and Department of Obstetrics and Gynecology, University of Nebraska Medical Center, Omaha, 68198-3255 NE USA

**Keywords:** Bayesian, Differential expression, Microarray, Next-generation sequencing

## Abstract

**Background:**

Recently, the Bayesian method becomes more popular for analyzing high dimensional gene expression data as it allows us to borrow information across different genes and provides powerful estimators for evaluating gene expression levels. It is crucial to develop a simple but efficient gene selection algorithm for detecting differentially expressed (DE) genes based on the Bayesian estimators.

**Results:**

In this paper, by extending the two-criterion idea of Chen et al. (Chen M-H, Ibrahim JG, Chi Y-Y. A new class of mixture models for differential gene expression in DNA microarray data. J Stat Plan Inference. 2008;138:387–404), we propose two new gene selection algorithms for general Bayesian models and name these new methods as the confident difference criterion methods. One is based on the standardized differences between two mean expression values among genes; the other adds the differences between two variances to it. The proposed confident difference criterion methods first evaluate the posterior probability of a gene having different gene expressions between competitive samples and then declare a gene to be DE if the posterior probability is large. The theoretical connection between the proposed first method based on the means and the Bayes factor approach proposed by Yu et al. (Yu F, Chen M-H, Kuo L. Detecting differentially expressed genes using alibrated Bayes factors. Statistica Sinica. 2008;18:783–802) is established under the normal-normal-model with equal variances between two samples. The empirical performance of the proposed methods is examined and compared to those of several existing methods via several simulations. The results from these simulation studies show that the proposed confident difference criterion methods outperform the existing methods when comparing gene expressions across different conditions for both microarray studies and sequence-based high-throughput studies. A real dataset is used to further demonstrate the proposed methodology. In the real data application, the confident difference criterion methods successfully identified more clinically important DE genes than the other methods.

**Conclusion:**

The confident difference criterion method proposed in this paper provides a new efficient approach for both microarray studies and sequence-based high-throughput studies to identify differentially expressed genes.

**Electronic supplementary material:**

The online version of this article (doi:10.1186/s12859-015-0664-3) contains supplementary material, which is available to authorized users.

## Background

In the past decade, high-throughput molecular technologies have gained great popularity in gene expression profiling due to their capability of producing thousands of measurements for each of the assayed samples. The microarray technology and next-generation sequencing are two widely used high-throughput technologies. Next-generation sequencing improves upon Sanger dideoxy sequencing so that the number of sequencing reactions in a single run can be in millions. For example, in Nature (2008), Bentley et al. [4] and Wang et al. [34] reported the DNA sequence of a Nigerian individual and an Asian individual, respectively. Ley et al. [18] analyzed the genome sequence of a tumor sample. One common scientific question addressed by these high-throughput experiments is to identify the genes with differential expression between two biological conditions. Although the high-throughput technologies offer us rich biological information, they are highly error-prone because many genes are monitored at the same time with a relatively small sample size. Bayesian methods provide a good solution to this problem because they synthesize all the data by borrowing information across different genes and produce more efficient estimators for evaluating the gene expressions. They include linear models in LIMMA [28] where empirical Bayesian methods were used to obtain stable results even with small sample size. A more detailed description of the Bayesian statistical methods for microarray studies can be found in Dudoit et al. [7], Pan [25], and Kuo et al. [15]. Other Bayesian methods for RNA-Seq studies using next generation sequencing were reviewed by Kvam et al. [16] and Soneson and Delorenzi [29].

Yu et al. [36] pointed out that most statistical methods for microarray studies examined the differential expressions by testing on the equality of means of the log-transformed intensities between the treatment and control, which may not be appropriate for data with complex structures (for example, a mixture normal distributions with multiple modes). They proposed a calibrated Bayes factor (CBF) method to evaluate the ratio of the full data marginal likelihood under the alternative hypothesis that a gene is differentially expressed (DE) relative to that for the null hypothesis that a gene is equivalently expressed (EE) between two biological conditions. Although their approach has the potential for handling data with more complicated distributions, the computational cost of their method may increase greatly with the complexity of the model.

Chen et al. [6] employed a class of mixture models with two components to fit the microarray data with two biological conditions. To evaluate the differential expressions for each gene, they proposed a gene selection algorithm, namely the two-criterion method. Specifically, they calculated a posterior probability that there is at least a two-fold change between the mean values of raw intensities under the two considered conditions. Then a gene is declared to be DE if the resulting posterior probability is large (say at least 0.7). Since the posterior probability is readily available once a Markov chain Monte Carlo sample is drawn from the posterior distribution, the gene selection algorithm proposed by them is quite easy to implement and computationally inexpensive. However, their approach does not consider general data distributions as that in the Bayes factor approach given by Yu et al. [36]. Assuming that the data under each biological condition follow a log-normal distribution as in [6], the mean value of raw intensities equals to exp(mean+variance/2) under each condition. Thus, the two-criterion method proposed by Chen et al. [6] that calculates the ratio of two means of the raw intensities depends on not only the difference between the two transformed means but also the difference between their variances. So, when the differences between the means and the differences between the variances are in opposite directions, the Chen et al. method may not be able to detect DE genes. Additionally, their paper neither provides a guidance on controlling the false discovery rate (FDR) nor carries out the performance comparison with other existing methods.

Our goal in this paper is to develop a simple but efficient gene selection algorithm so that it is not only computationally efficient, but also flexible in handling data with a complicated distribution as in Yu et al. [36]. We redevelop the two-criterion method proposed by Chen et al. [6] and construct two new gene selection algorithms for general Bayesian models. One is based on the differences between means and the other is based on both mean differences and variance differences. To differentiate the method proposed in Chen et al. [6], we name our methods as confident difference criterion methods and the two proposed confident difference criterion methods in this paper as Methods I and II. We show that the Method I, which compares the mean expressions from different conditions, is equivalent to the calibrated Bayes factor approach [36] when the raw intensities from two different biological conditions follow log-normal distributions with equal variance. We also address the multiple comparisons issue with a control of the false discovery rate. We further apply the proposed method to carry out analyses of microarray data with more than two conditions as well as sequence-based RNA data.

## Method

### Model for microarray data

We assume that the data, denoted by *D*_*obs*_, have already been preprocessed with appropriate transformation and normalization. Let T be the total number of biological conditions in the study. The data may contain two biological conditions (*T*=2) or multiple biological conditions (*T*>2). The common analytical objective is to detect differentially expressed (DE) genes across different biological conditions.

Let *x*_*gtk*_ denote the preprocessed expression intensity of the *g* ^*th*^ gene in the *k* ^*th*^ sample under the *t* ^*th*^ condition for *t*=1,⋯,T. There are a total of G genes with sample size *n*_*gt*_ under condition *t*. Thus, the data on gene *g* under each condition can be summarized using a vector: $\mathbf {X}_{\textit {gt}}=(x_{gt1},\ldots,x_{gtn_{\textit {gt}}})\phantom {\dot {i}\!}$. We assume that the intensity, *x*_*gtk*_, *k*=1,⋯,*n*_*gt*_, *t*=1,2,⋯,T, follows a normal distribution $\mathcal {N}\left (\mu _{\textit {gt}}, \sigma ^{2}_{\textit {gt}}\right)$ independently. The parameters *μ*_*gt*_ and $\sigma ^{2}_{\textit {gt}}$ denote the mean and variance of the intensities of gene *g* under condition *t*, respectively. We write the mean intensities as *μ*_*gt*_ = *μ*_*g*_ + *δ*_*gt*_/*T*, *t* = 1,…,T, where $\sum ^{T}_{t=1}\delta _{\textit {gt}}\,=\,0$. For simplicity, we set $\delta _{g1}=-\sum ^{T}_{t=2} \delta _{\textit {gt}}$ under the first biological condition. We note that *μ*_*g*_ defines the overall mean of the intensities across all biological conditions, and *δ*_*gt*_/*T* measures the difference in the mean intensity under biological condition *t* from the overall mean. In a microarray study with two biological conditions (*T*=2), the mean intensities *μ*_*g*1_ and *μ*_*g*2_ are written as *μ*_*g*1_=*μ*_*g*_−*δ*_*g*_/2 and *μ*_*g*2_=*μ*_*g*_+*δ*_*g*_/2, respectively. When a gene is DE, we expect that the distributions of the data differ at least under two biological conditions.

#### Hierarchial prior distributions

Noninformative conditionally conjugate priors are specified for all parameters. Specifically, we assume that the mean parameters $\mu _{g} \sim \mathcal {N}(0,\tau ^{2})$ and $\delta _{\textit {gt}} \sim \mathcal {N} (0, \omega ^{2})$ for *t*>1 and any *g*, and the variance parameters $\sigma ^{2}_{\textit {gt}} \sim \mathcal {IG}(a_{t},b_{t})$. We set the variance parameters *τ* ^2^ and *ω* ^2^ in the normal priors to be 100 to obtain relatively noninformative priors. The shape parameter *a*_*t*_ in the inverse gamma prior is set to be 2, so that the prior mean of $\sigma _{\textit {gt}}^{2}$ equals *b*_*t*_. We further let the scale parameter *b*_*t*_ follow a conditionally conjugate gamma prior with $b_{t} \sim \mathcal {G}(c,d)$, where the hyperparameter *c* is specified as 1 and the hyperparameter $d \sim \mathcal {IG}(a_{d},b_{d})$, in which *a*_*d*_ and *b*_*d*_ are both set to be 0.01 in the simulation study and 1 in the real data analysis. Our hierarchical priors for the variance parameters, which are often difficult to estimate, allow for borrowing the information across genes via $b_{t} \sim \mathcal {G}(c,d)$ as well as biological conditions via $d \sim \mathcal {IG}(a_{d},b_{d})$. We intend to specify a noninformative inverse-gamma prior for the parameter *d*. The value of “1" was specified for both *a*_*d*_ and *b*_*d*_ in the real data analysis since the real data had a smaller sample size than the simulated data in the simulation study. These values of the hyperparameters still led to noninformative priors since the prior mean and variance of *d* do not exist. However, these values allowed us to borrow a little but not too much information across different biological conditions under comparison.

#### Conditional posterior distributions

Let $\bar {x}_{\textit {gt}}$ denote the average intensities of gene *g* under condition *t* and also let the vector $\bar {\mathbf {X}}_{g}=\{\bar {x}_{g1},\cdots,\bar {x}_{\textit {gT}}\}$ denote the average intensities for gene *g*. Then, $\bar {\mathbf {X}}_{g}$ follows a multivariate normal distribution with $\bar {\mathbf {X}}_{g} \sim \mathcal {N}(\mathbf {A}\mathbf {\Theta }_{g},\mathbf {\Sigma }_{g})$, where **Θ**_*g*_=(*μ*_*g*_,*δ*_*g*2_,⋯,*δ*_*gT*_)^′^ is a column vector of size *T*, and **Σ**_*g*_ is a diagonal matrix of size *T*×*T* with the *t* ^*th*^ diagonal element $(\mathbf {\Sigma }_{g})_{t,t}=\sigma ^{2}_{\textit {gt}}/n_{\textit {gt}}$. Here **A** is a *T*×*T* matrix, in which all elements in the first column equals one, i.e., **A**_*t*1_=1 for *t*=1,⋯,*T*, all but the first element in the first row equals −1/*T*, i.e., **A**_1*t*_=−1/*T* for *t*=2,⋯,*T*, all but the first diagonal element equals 1/*T*, i.e., **A**_*t*,*t*_=1/*T* for *t*=2,⋯,*T*, and all other elements equal zero. Since the parameters in **Θ**_*g*_ independently follow normal prior distributions, then $\mathbf {\Theta }_{g} \sim \mathcal {N}(\mathbf {0},\mathbf {\Sigma }_{0})$, where **0** is a vector of size *T* containing all zero’s and **Σ**_0_ is a diagonal matrix with the first diagonal element (**Σ**_0_)_1,1_=*τ* ^2^ and all other diagonal elements equal to *ω* ^2^, i.e., (**Σ**_0_)_*t*,*t*_=*ω* ^2^ for *t*=2,⋯,*T*. Therefore, the conditional posterior distribution of **Θ**_*g*_ is a multivariate normal distribution with **Θ**_*g*_∼*N*(**U**_*g*_,**B**_*g*_), where the inverse of the variance matrix $\mathbf {B}_{g}^{-1}=\left (\mathbf {A}'\mathbf {\Sigma }_{g}^{-1} \mathbf {A}+\mathbf {\Sigma }_{0}^{-1}\right)$, and the mean vector $\mathbf {U}_{g}= \mathbf {B}_{g} \mathbf {A}' \mathbf {\Sigma }_{g}^{-1} \bar {\mathbf {X}}_{g}$. The conditional posterior distribution of the variance parameter $\sigma _{\textit {gt}}^{2}$ is an inverse-gamma distribution with $\sigma _{\textit {gt}}^{2} \sim \mathcal {IG}\left (a_{t}+\frac {1}{2}n_{\textit {gt}}, b_{t}+\frac {1}{2}\sum _{k=1}^{n_{\textit {gt}}}\left (x_{\textit {gtk}}-\mu _{\textit {gt}}\right)^{2}\right)$. The conditional posterior density of the hyperparameter *b*_*t*_ is given by $g\left (b_{t}|\sigma _{1t}^{2},\cdots, \sigma _{\textit {Gt}}\right) \propto b_{t}^{(\mathrm {G} a_{t}/2+c)}\exp \left (-\frac {b_{t}}{2}\sum {\sigma _{\textit {gt}}^{-2}}\right)\times \left (\sum _{t'\ne t} b_{t}'+b_{t}+b_{d}\right)^{\mathrm {T} c+a_{d}}$. Consequently, we can apply the Gibbs sampling algorithm to sample the parameters *b*_*t*_, $\sigma ^{2}_{\textit {gt}}$ and **Θ**_*g*_ in turn from their respective conditional posterior distributions using the following steps: (1) sample *b*_*t*_ for each condition *t* from its posterior density function $g(b_{t}|\sigma _{1t}^{2},\cdots, \sigma _{\textit {Gt}})$ via the Metropolis-Hastings algorithm; (2) sample $\sigma ^{2}_{\textit {gt}}$ given *b*_*t*_ and *μ*_*gt*_ for each *g* and *t* from its inverse gamma posterior distribution with updated parameters $a_{t}+\frac {1}{2}n_{\textit {gt}}$ and $b_{t}+\frac {1}{2}\sum _{k=1}^{n_{\textit {gt}}}(x_{\textit {gtk}}-\mu _{\textit {gt}})^{2}$; (3) sample *Θ*_*g*_ given $\sigma ^{2}_{\textit {gt}}$ for all *g* from their conditional multivariate normal posterior distribution, and calculate the *μ*_*gt*_ based on the sampled values of *Θ*_*g*_.

### Model for sequence-based data

Let $\phantom {\dot {i}\!}\textit {\textbf {Y}}_{\textit {gt}}=(y_{gt1},\cdots y_{gtn_{\textit {gt}}})$ denote all *n*_*gt*_ observed counts of the expressed tags of gene *g* under condition *t* for *g*=1,⋯,G and *t*=1,⋯,T. We assume that *y*_*gkt*_ follows a negative binomial distribution, which is commonly used for the count data with overdispersion [2, 26]. Specifically, we assume *y*_*gtk*_ follows $\mathcal {NB}\left (\phi _{t},\frac {m_{\textit {tk}}\lambda _{\textit {gt}}}{\phi _{t}+m_{\textit {tk}}\lambda _{\textit {gt}}}\right)$, with mean *m*_*tk*_ *λ*_*gt*_ and variance $m_{\textit {tk}}\lambda _{\textit {gt}}\left (1+m_{\textit {tk}}\lambda _{\textit {gt}}\phi _{t}^{-1}\right)$. We set *m*_*tk*_ to be the library size of the *k* ^*th*^ sample under the *t* ^*th*^ condition, which is the sum of all counts from this library. The dispersion parameter *ϕ*_*t*_ is assumed to be positive, accounting for potential over-dispersion in the data. When the dispersion parameter *ϕ*_*t*_ gets extremely large, the value of $\phi _{t}^{-1}$ approaches to zero, and the negative binomial distribution becomes a Poisson distribution with a mean value of *m*_*tk*_ *λ*_*gt*_. DE genes are expected to have different *λ*_*gt*_’s under different biological conditions.

#### Hierarchical prior distributions

We assume that each dispersion parameter *ϕ*_*t*_ follows a gamma distribution, $\phi _{t} \sim \mathcal {G}(\alpha _{\phi }, \beta _{\phi })$ independently over *t* and its scale parameter *β*_*ϕ*_ follows an inverse gamma distribution with $\beta _{\phi } \sim \mathcal {IG}(\zeta _{\phi },\eta _{\phi })$. We also assume that each gene expression parameter *λ*_*gt*_ follows an inverse gamma distribution with $\lambda _{\textit {gt}} \sim \mathcal {IG}(\alpha _{\lambda _{t}},\beta _{\lambda _{t}})$, where the scale parameter $\beta _{\lambda _{t}} \sim \mathcal {G}(\zeta _{\lambda },\eta _{\lambda })$. In our simulation studies, we set all the hyperparameters $\{\alpha _{\phi },\zeta _{\phi }, \eta _{\phi },\alpha _{\lambda _{t}},\zeta _{\lambda },\eta _{\lambda }\}\phantom {\dot {i}\!}$ to be one.

#### Conditional posterior distributions

Since a negative binomial distribution can be written as a Poisson-gamma distribution, we can rewrite the distribution of *y*_*gtk*_ as $y_{\textit {gtk}} \sim \mathcal {P}oi(\theta _{\textit {gtk}})$, and $\theta _{\textit {gtk}} \sim \mathcal {G}\left (\phi _{t},m_{\textit {tk}} \lambda _{\textit {gt}} \phi _{t}^{-1}\right)$. Then we can derive all the conditional posterior distributions for all of the parameters. Specifically, the conditional posterior distribution of *θ*_*gtk*_ is a gamma distribution with $\theta _{\textit {gtk}} \sim \mathcal {G}\left (y_{\textit {gtk}}+\phi _{t},\left [1+\frac {\phi _{t}}{m_{\textit {tk}}\lambda _{\textit {gt}}}\right ]^{-1}\right)$, the kernel of the conditional posterior density of *ϕ*_*t*_ is given by $\prod _{\textit {gk}} \left [\frac {\phi _{t}^{\phi _{t}}}{\Gamma (\phi _{t})} \left (\frac {\theta _{\textit {gtk}}}{m_{\textit {tk}}\lambda _{\textit {gt}}}\right)^{\phi _{t}} \exp \left (-\frac {\theta _{\textit {gtk}}}{m_{\textit {tk}}\lambda _{\textit {gt}}}\phi _{t}\right) \right ] \exp \left (-\frac {\phi _{t}}{\beta _{\phi }}\right)\phi _{t}^{\alpha _{\phi }-1} I(\phi _{t}>0)$, the conditional posterior distribution of *λ*_*gt*_ is $\mathcal {IG}\left (\sum _{k}\phi _{t}+\alpha _{\lambda _{t}}, \beta _{\lambda _{t}}+\sum _{k}\frac {\theta _{\textit {gtk}}\phi _{t}}{m_{\textit {tk}}}\right)$, and the hyperparameters *β*_*ϕ*_, and $\beta _{\lambda _{t}}$ respectively have the conditional posterior distributions: $\beta _{\phi } \sim \mathcal {IG}\left (T\alpha _{\phi }+\zeta _{\phi }, \sum _{t}\phi _{t}+\eta _{\phi }\right)$, and $\beta _{\lambda _{t}} \sim \mathcal {G}\left (G\alpha _{\lambda }+\zeta _{\lambda },1/\left (1/\eta _{\lambda }+\sum _{g} 1/\lambda _{\textit {gt}}\right)\right)$. Let ***θ***_*t*_ denote a set containing all *θ*_*gtk*_’s and ***λ***_*t*_ as a set containing all *λ*_*gt*_’s for each condition *t*. We use the Gibbs sampling algorithm to sample parameters $\phantom {\dot {i}\!}\{\boldsymbol {\theta }_{t}, \boldsymbol {\lambda }_{t}, \beta _{\lambda _{t}}\}$, ∀*t*, and *β*_*ϕ*_ from their conditional posterior distributions. The conditional posterior distribution of *ϕ*_*t*_ does not have a known distribution form. These parameters are sampled using the Metropolis-Hastings sampling algorithm from their conditional posterior distributions.

## Confident difference criterion

### Preliminary

The confident difference criterion method was extended from the two-criterion method, which was firstly proposed by Ibrahim et al. [13] to detect DE genes for microarray studies with two biological conditions. In this two-criterion method, the fold change between two conditions was defined as $\xi _{g}=\exp \left (\mu _{g2}+0.5\sigma _{g2}^{2}-\mu _{g1}\right.-$ $\left.0.5\sigma _{g1}^{2}\right)$, and the posterior probabilities of having at least two fold changes between two conditions, denoted as *γ*_*g*1_=*P* *r*(*ξ*_*g*_>2|*D*_*obs*_) and *γ*_*g*2_=*P* *r*(*ξ*_*g*_<1/2|*D*_*obs*_), were evaluated on each gene to quantify the evidence of its differential expression. A gene is declared to be DE genes if the calculated posterior probabilities *γ*_*g*1_ or *γ*_*g*2_ are sufficiently large. The two-criterion method is easy to compute and provides good false positive and false negative rates [6] for identifying DE genes from microarray studies with two biological conditions. However, the posterior probability *γ*_*g*_ defined in this confident difference criterion method does not account for the posterior variability of the fold change, and may not work well for the data with multiple conditions due to the potential multiple comparisons problem since only two conditions can be compared at a time.

In this section, we will develop confident difference criterion using a similar idea of the existing two-criterion method to compare mean expressions (Method I) after taking into account the posterior variability of the mean intensity parameters for the microarray data with two biological conditions. Then we extend the newly developed confident difference criterion method for the microarray data with multiple biological conditions. Furthermore, we will develop another version of the confident difference criterion method to compare both means and variances of the expressions (Method II) for the microarray data. Finally, we extend the confident difference criterion method for comparing mean differential expressions of microarray data (Method I) to the analysis of RNA-Seq data (Method I).

### Confident difference criterion for the comparison between mean expressions for the microarray data

#### Microarray study with two conditions

For a study with two biological conditions, *μ*_*g*2_−*μ*_*g*1_ quantifies the difference in the mean intensities of gene *g* between the two conditions and its conditional posterior distribution follows a normal distribution. We define the posterior probability as

(1) $$  \gamma_{g}=Pr\left(\frac{|\mu_{g2}-\mu_{g1}|}{\sigma_{\mu_{g2}-\mu_{g1}}}>2 \Bigg| D_{obs}\right),  $$

where $\sigma _{\mu _{g2}-\mu _{g1}}$ is the posterior standard deviation of *μ*_*g*2_−*μ*_*g*1_. Then we select a cutoff value *γ*_0_ (0<*γ*_0_<1) and declare a gene to be DE if its posterior probability *γ*_*g*_ is greater than the cutoff value *γ*_0_.

Note that the choice of *γ*_0_ reflects how strong the evidence is for declaring DE genes. When a larger value is specified for *γ*_0_, fewer genes will be selected to be DE. In the two-criterion method, Chen et al. [6] recommended to use a large cutoff value (ranging between 0.7 and 0.9) because they did not adjust for the posterior variability of the fold change when comparing the gene intensities between the two conditions. After adjusting for the posterior variability, *γ*_*g*_ in (1) is quite different than the corresponding posterior probability under the two-criterion method of Chen et al. [6], as shown in the following proposition.

##### **Proposition1**.

Assume that the difference in the mean intensities, *μ*_*g*2_−*μ*_*g*1_, follows a normal distribution. The proposed confident difference criterion method ensures that if *γ*_*g*_≥*γ*_0_, then the maximum value of the posterior probabilities for the difference *μ*_*g*2_−*μ*_*g*1_ being larger or smaller than zero, i.e., max{*P* *r*(*μ*_*g*2_−*μ*_*g*1_>0 |*D*_*obs*_), *P* *r*(*μ*_*g*2_−*μ*_*g*1_<0 |*D*_*obs*_)}, is at least *Φ*(2−*Φ* ^−1^(1+*Φ*(−2)−*γ*_0_)) for *γ*_0_>*Φ*(−2), where *Φ* and *Φ* ^−1^ denote the cumulative distribution function (cdf) and the inverse cdf of the standard *N*(0,1) distribution, respectively. The detailed proof is presented in Additional file 1.

**Fig. 1:**
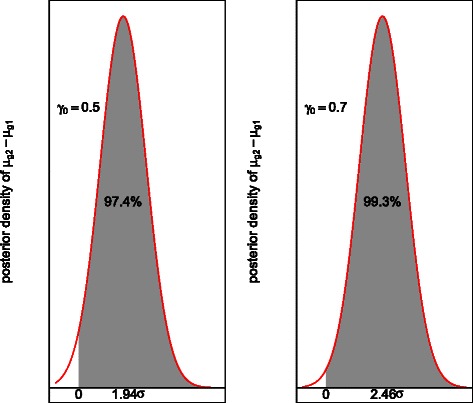
Graphical illustration of the confident difference criterion method. The figure on the left panel and the right panel uses *γ*_0_=0.5 and *γ*_0_=0.7 separately. The *μ*_*g*2_−*μ*_*g*1_ measures the difference in the mean intensities of gene *g* between the two conditions. Both figures are drawn based on an assumption that the posterior mean of *μ*_*g*2_−*μ*_*g*1_ are positive. The shaded area in both figures measures the posterior probability for having a positive difference *μ*_*g*2_−*μ*_*g*1_

We note that the maximum value of the posterior probabilities for the difference *μ*_*g*2_−*μ*_*g*1_ being larger or smaller than zero measures a Bayesian p-value. Figure 1 shows a graphical presentation of the Proposition 1 with *γ*_0_ chosen to be 0.5 and 0.7, respectively. For example, we use $\xi _{\mu _{g2}-\mu _{g1}}$ to denote the posterior mean value of the difference *μ*_*g*2_−*μ*_*g*1_. When *γ*_0_=0.5 and assuming that the posterior mean value $\xi _{\mu _{g2}-\mu _{g1}}>0$, $\xi _{\mu _{g2}-\mu _{g1}}$ is at least $1.94 \sigma _{\mu _{g2}-\mu _{g1}}$ away from zero. The maximum value of the posterior probabilities for the difference *μ*_*g*2_−*μ*_*g*1_ being larger or smaller than zero, max{*P* *r*(*μ*_*g*2_−*μ*_*g*1_>0 |*D*_*obs*_), *P* *r*(*μ*_*g*2_−*μ*_*g*1_<0 |*D*_*obs*_)}, will be at least *Φ*(2−*Φ* ^−1^(1+*Φ*(−2)−0.5))=97.4 *%*. Therefore, we recommend to use a smaller cutoff value than the previous two-criterion method [6] when using (1) for identifying DE genes. Possible choices of the cutoff value *γ*_0_ may range from 0.4 to 0.7.

#### Connection with the CBF method for microarry study with two conditions

For a microarray study with two biological conditions, we assume that the preprocessed expression intensity from each biological condition follows a normal distribution with $x_{\textit {gtk}} \sim N\left (\mu _{\textit {gt}},\sigma _{\textit {gt}}^{2}\right)$, and the parameters follow the prior distribution specified in the aforementioned Model for microarray data subsection. For simplicity, we assume that the equal number of intensities are observed from the same gene under different conditions, and they share the same known variance, i.e., *n*_*g*1_=*n*_*g*2_=*n*_*g*_ and $\sigma ^{2}_{g1}=\sigma ^{2}_{g2}={\sigma ^{2}_{g}}$. The proposed confident difference criterion method is used to detect differentially expressed genes. Alternatively, we can also apply the CBF method for the data analysis. To detect differentially expressed genes, we test on the null hypothesis that the mean intensities are equal (*μ*_*g*1_=*μ*_*g*2_) against the alternative hypothesis that the mean intensities are unequal (*μ*_*g*1_≠*μ*_*g*2_) between the two biological conditions. We use the same prior distributions as that in the confident difference criterion method under the alternative hypothesis, and similar prior distributions for the parameters under the null hypothesis. With simple algebra, we can show that the proposed confident difference criterion method for comparing the mean intensities between two biological conditions agrees with the CBF method under the condition stated in the following Proposition.

##### **Proposition2**.

The confident difference criterion method comparing the mean intensities between the two biological conditions with a cut-off value of *γ*_0_ agrees with the CBF method for the hypotheses testing on whether the mean intensities are equal between the two biological conditions with a cut-off value of *C*_0_ if $\Phi \left (2+E_{g}^{\ast }\right)-\Phi \left (-2+E_{g}^{\ast }\right)=1-\gamma _{0}$, where $|E_{g}^{\ast }|= \left [ \log \left (\frac {n_{g}\omega ^{2}}{2{\sigma ^{2}_{g}}}+1\right) -2\log (C_{0})\right ]^{\frac {1}{2}}$, provided that the cutoff value *C*_0_ is chosen so that the argument in the square root expression is non-negative. The detailed proof is presented in Additional file 1.

#### Microarray study with multiple conditions

The confident difference criterion method can be extended to microarray studies with multiple biological conditions. Our primary interest of the study is to identify genes that have differential expressions at least between two biological conditions. Therefore, we define a quadratic form to quantify the differences in the gene expression intensities across different biological conditions, and conduct an overall test to determine whether the mean intensities are different at least under two biological conditions on each gene.

Considering the first biological condition as a reference condition, we define a column vector ***Δ***_*μ*,*g*_=(*μ*_*g*2_−*μ*_*g*1_,⋯,*μ*_*gT*_−*μ*_*g*1_)^′^ to measure the difference in the mean intensities between each non-reference biological condition and the reference condition. Let $\phantom {\dot {i}\!}\Sigma _{\boldsymbol {\Delta }_{\mu, g}}$ be the posterior covariance matrix of ***Δ***_*μ*,*g*_. We then propose the quadratic form, $\boldsymbol {\Delta }_{\mu, g}'\Sigma ^{-1}_{\boldsymbol {\Delta }_{\mu, g}}\boldsymbol {\Delta }_{\mu, g}$, to quantify the differential gene expressions for all non-reference biological conditions compared to the reference condition. Under the null hypothesis that gene *g* is not DE, the quadratic form follows a chi-square distribution with *d* *f*=*T*−1 when ***Δ***_*μ*,*g*_ is assumed to follow a multivariate normal distribution. We note that the multivariate normality holds asymptotically when the sample size is large. We choose an integer, denoted as *C*, which is closest to the 95 ^*th*^ percentile of the chi-square distribution. For example, for a microarray study with three biological conditions (i.e., *T*=3), the corresponding *C* value equals 6. Similar to (1), we compute the posterior probability

(2) $$  \gamma_{g}=Pr\left(\boldsymbol{\Delta}_{\mu, g}'\Sigma^{-1}_{\boldsymbol{\Delta}_{\mu, g}} \boldsymbol{\Delta}_{\mu, g}>C|D_{obs}\right),  $$

and declare gene *g* to be DE if *γ*_*g*_≥*γ*_0_.

### Confident difference criterion for comparison of both means and variances of expression for microarray data

We note that the confident difference criterion method proposed so far only evaluates the differences in mean intensities. Recall that the Bayes factor approach in Yu et al. [36] is more desirable since it compares both means and variances of the intensities for each gene. Assume that the means and variances are equally important. An appropriate quadratic form can be constructed to quantify the overall difference between both the means and the variances under different conditions on each gene. Since the posterior distribution of $\sigma _{\textit {gt}}^{2}$’s is typically skewed, a stabilization transformation of the variance $\sigma _{\textit {gt}}^{2}$ is required. Let *q*(.) denote a one-to-one transformation function. The differences in both means and transformed variances of the intensities across different conditions can be summarized in a quadratic form given by

(3) $$ Q_{g}=\boldsymbol{\Delta}_{\boldsymbol{\mu},\boldsymbol{\sigma},g}' \Sigma^{-1}_{\boldsymbol{\Delta}_{\boldsymbol{\mu},\boldsymbol{\sigma}, g}} \boldsymbol{\Delta}_{\boldsymbol{\mu},\boldsymbol{\sigma}, g},   $$

where $\boldsymbol {\Delta }_{\boldsymbol {\mu }, \boldsymbol {\sigma },g}=\left (\mu _{g2}-\mu _{g1},\cdots, \mu _{\textit {gT}}-\mu _{g1}, q\left (\sigma ^{2}_{g2}\right)-q \left (\sigma ^{2}_{g1}\right),\cdots, q\left (\sigma ^{2}_{\textit {gT}}\right)-q\left (\sigma ^{2}_{g1}\right)\right)'$ is a column vector of length 2*T*−2 containing the differences in both means and transformed variances of the intensities. The covariance matrix $\Sigma _{\boldsymbol {\Delta }_{\boldsymbol {\mu },\boldsymbol {\sigma },g}}\phantom {\dot {i}\!}$ is the posterior covariance matrix of ***Δ***_***μ***,***σ***,*g*_. Since *q*(·) is a one-to-one transformation function, then we have $ \sigma ^{2}_{\textit {gt}}=\sigma ^{2}_{gt'}$ if and only if $ q\left (\sigma ^{2}_{\textit {gt}}\right)=q\left (\sigma ^{2}_{gt'}\right)$ for *t*≠*t* ^′^. Thus, the same *q*(·) function has to be used across all the *T* treatment groups. The primary reason for introducing the transformation function *q*(·) is to make the distribution of $q\left (\sigma ^{2}_{\textit {gt}}\right)$ more normal.

Similar to (2), we compute the posterior probability *γ*_*g*_=*P* *r*(*Q*_*g*_>*C*|*D*_*obs*_), where *C* is chosen to be an integer, which is closest to the 95 ^*th*^ percentile of the chi-square distribution with *d* *f*=2*T*−2. For example, *C* will be chosen to be 9 when *T*=2, and 13 when *T*=3. In this paper, we consider the negative cube root transformation on the variance parameters $\sigma ^{2}_{\textit {gt}}$’s. The cube root transformation, also known as Wilson-Hilferty transformation, was derived by Wilson and Hilferty [35] to transform a chi-square variate to be approximately normally distributed. In the proposed gene selection algorithm below, the cutoff value *γ*_0_ will be automatically determined to control the false discovery rate to be less than a targeted level.

### Confident difference criterion for sequence-based data

As discussed in the Model for sequence-based data subsection, the parameter *λ*_*gt*_ quantifies the expression level of gene *g* under condition *t*. The differences in *λ*_*gt*_’s measure the relative differential expressions of gene *g* between the conditions. Note that the *λ*_*gt*_’s likely have small values and their posterior distributions may be skewed. Therefore, we apply a log transformation on *λ*_*gt*_’s and use the differences in log*λ*_*gt*_’s to quantify the differential gene expressions among different biological conditions. Similar to the confident difference criterion method for microarray data, we propose the confident difference criterion method for the sequence-based data with two biological conditions as follows. We first compute

(4) $$ \gamma_{g}=Pr\left(\frac{|\log(\lambda_{g2})-\log(\lambda_{g1})|} {\sigma_{\log(\lambda_{g2})-\log(\lambda_{g1})}}>2|D_{obs}\right),  $$

where $\sigma _{\log (\lambda _{g2})-\log (\lambda _{g1})}$ is the posterior standard deviation for the difference log(*λ*_*g*2_)− log(*λ*_*g*1_). We then declare gene *g* to be DE if *γ*_*g*_≥*γ*_0_, where 0<*γ*_0_<1 is a predetermined credible level.

When the sequence-based data are collected from multiple conditions, the first biological condition will be considered as the reference condition and a column vector ***Δ***_*λ*,*g*_=(log(*λ*_*g*2_)− log(*λ*_*g*1_), log(*λ*_*g*3_)− log(*λ*_*g*1_),⋯, log(*λ*_*gT*_)− log(*λ*_*g*1_))^′^ contains the differences in the log scaled expression values between the non-reference conditions and the reference condition. Let ${\Sigma }_{\boldsymbol {\Delta }_{\lambda,g}}\phantom {\dot {i}\!}$ denote the posterior covariance matrix of ***Δ***_*λ*,*g*_. Accordingly, the confident difference criterion method is defined as $\gamma _{g}=Pr\left ({\boldsymbol {\Delta }}_{\lambda,g}'\mathbf {\Sigma }^{-1}_{{\boldsymbol {\Delta }}_{\lambda,g}} {\boldsymbol {\Delta }}_{\lambda,g}>C_{\lambda }|D_{\textit {obs}}\right)$, where *C*_*λ*_ is an integer, which is closest to the 95 ^*th*^ percentile of the chi-square distribution with *d* *f*=*T*−1. Again, we declare gene *g* to be DE if *γ*_*g*_≥*γ*_0_, where 0<*γ*_0_<1.

From the Model for sequence-based data subsection, we see that the variance of the observed count *y*_*gtk*_ is $m_{\textit {tk}}\lambda _{\textit {gt}} \left (1+ m_{\textit {tk}} \lambda _{\textit {gt}} \phi ^{-1}_{t}\right)$, which is a function of *λ*_*gt*_ and *ϕ*_*t*_, for the sequence data. Since *ϕ*_*t*_ does not depend on *g*, it is sufficient to compare the mean expressions under different conditions for determining DE genes for the sequence data.

## False discovery rate and gene selection algorithm

The proposed confident difference criterion methods calculate the value of *γ*_*g*_ on each gene, whose magnitude reflects the evidence of differential expression. When *γ*_*g*_ is large enough, the gene will be declared to be DE. It is of great importance to determine how to choose the cutoff value *γ*_0_.

We adopt the approach proposed by Tadesse et al. [31] to select the cutoff value *γ*_0_ for controlling the Bayesian FDR. Let *V* denote the number of incorrect decisions by identifying EE genes as DE genes and let *R* be the number of identified DE genes. Then the positive false discovery rate defined by Storey [30] is given by $pFDR=E\left (\frac {V}{R}|R>0\right)$.

We need to test the hypotheses of *H*_0*g*_: gene *g* is EE versus *H*_1*g*_: gene *g* is DE on each gene. We assume that all genes have the same probability of being EE, and DE, respectively, i.e., *P* *r*(*H*_0*g*_)’s are equal for all genes, and *P* *r*(*H*_1*g*_)’s are equal for all genes. Therefore, the *γ*_*g*_’s are independently and identically distributed. Following Tadesse et al. [31], the Bayesian FDR *b* *F* *D* *R*(*γ*_0_) when using a cutoff value of *γ*_0_ for the confident difference criterion method is defined as

(5) $$ bFDR(\gamma_{0})=\frac{1}{Pr(R>0)}\times \frac{Pr\left(\gamma_{g} \ge \gamma_{0}|H_{0g}\right)Pr(H_{0g})}{Pr(\gamma_{g} \ge \gamma_{0})},   $$

and *P* *r*(*γ*_*g*_≥*γ*_0_)=*P* *r*(*γ*_*g*_≥*γ*_0_|*H*_0*g*_)*P* *r*(*H*_0*g*_)+*P* *r*(*γ*_*g*_≥*γ*_0_|*H*_1*g*_)*P* *r*(*H*_1*g*_). To estimate the *FDR*, we need to compute *P* *r*(*γ*_*g*_≥*γ*_0_|*H*_0*g*_), *P* *r*(*γ*_*g*_≥*γ*_0_|*H*_1*g*_) and *P* *r*(*H*_1*g*_). Note that gene *g* can be classified into DE or EE depending on whether *γ*_*g*_≥*γ*_0_. We reuse the data information and specify the prior probability *P* *r*(*H*_1*g*_) as the proportion of genes classified as DE. Denote the total number of identified DE genes as *n*_*D*_. Then the probability of a gene being DE will be *P* *r*(*H*_1*g*_)=*n*_*D*_/*G*. Additionally, we estimate the true parameters in the gene expression data distributions from DE or EE genes as the posterior means of the corresponding parameters from the identified DE and EE genes, respectively. An algorithm using the posterior samples from DE or EE genes to estimate the aforementioned probabilities *P* *r*(*γ*_*g*_≥*γ*_0_|*H*_0*g*_), *P* *r*(*γ*_*g*_≥*γ*_0_|*H*_1*g*_) and *b* *F* *D* *R*(*γ*_0_) is given as follows.

- Split the genes into two subsets containing *n*_*E*_ EE genes (*EEGENE*) with calculated *γ*_*g*_<*γ*_0_ and *n*_*D*_ DE genes (*DEGENE*) with *γ*_*g*_≥*γ*_0_.
- Note that a DE gene can be either up or down regulated under some condition compared to the reference condition in terms of means or variances of the expression values for microarray experiment or in terms of mean gene expressions for sequence-based experiment. Accordingly, the DE genes will be further split into a series of gene subsets based on the pattern of parameters in comparisons under different biological conditions. For example, in a microarray study with three biological conditions and the mean gene expressions are in comparison. Consider the first condition as the reference condition. The DE genes can be classified into four subsets: (i) genes with lower mean gene expressions under both conditions 2 and 3; (ii) genes with lower mean gene expressions under condition 2 but higher mean gene expressions under condition 3; (iii) genes with higher mean gene expressions under condition 2 but lower mean gene expressions under condition 3; and (iv) genes with higher mean gene expressions under both conditions 2 and 3. We denote these subsets of DE genes as *D*_*ℓ*_,*ℓ*=1,⋯,*L*, where the number of subsets *L*=2^*T*−1^ for the microarray study when only mean parameters are in comparison or the sequenced-based study; and *L*=4^*T*−1^ for the microarray study when both mean and variance parameters are in comparison. We also denote the number of genes in the *D*_*ℓ*_ DE gene subsets as *n*_*Dℓ*_.
- For EE genes identified in previous steps, the same data distributions will be considered for the gene expression data from different biological conditions. Hierarchical priors similar to those proposed previously in the Model for microarray data section and the Model for sequence-based data subsection will be augmented separately for microarry data or sequence-based data. The posterior mean of each parameter defined in the distribution of the gene expression data will be calculated. The true parameters in the gene expression data distribution will be estimated using the average value of posterior mean of corresponding parameters from all EE genes. For all identified DE genes, the Markov chain Monte Carlo (MCMC) sampling values from previous steps when implementing the proposed confident difference criterion method will be used for calculating the posterior means of the parameters defined in the gene expression data distribution. For each differential gene expression pattern *ℓ*, the actual value of each parameter in the gene expression data distribution will be estimated using the average value of its posterior means across all genes in the subset *D*_*ℓ*_ with this DE pattern.
- Using the estimated values for the parameters in the gene expression data, the data will be simulated for *κ*×*G* genes (say *κ*=0.1), among which *κ* *n*_*E*_ EE genes and $\kappa n_{D_{\ell }}, \ell =1,\dots,L$ DE genes with a pattern of differential gene expression observed in the DE gene subset *D*_*ℓ*_, respectively.
- The posterior probability *γ*_*g*_ will be calculated for each gene based on the simulated data. Depending on whether *γ*_*g*_≥*γ*_0_, the gene in the simulated data will also be claimed to be DE or EE.
- Denote the total number of identified DE and EE genes from the simulated data as *m*_*D*_ and *m*_*E*_. Then the probability for a EE genes claimed to be DE, *P* *r*(*γ*_*g*_≥*γ*_0_|*H*_0*g*_), will be estimated as $Pr(\gamma _{g} \ge \gamma _{0}|H_{0g})=\frac {m_{E}}{\kappa n_{E}}$; and the probability for a DE genes claimed to be DE, *P* *r*(*γ*_*g*_≥*γ*_0_|*H*_1*g*_), will be estimated as $Pr(\gamma _{g} \ge \gamma _{0}|H_{1g})=\frac {m_{D}}{\kappa n_{D}}$. Using (5), the estimated Bayesian FDR equals $\widehat {bFDR}=\frac {m_{E}}{m_{D}+m_{E}}$.

Note that steps (4) to (6) provide a predictive approach to estimate bFDR when a certain value *γ*_0_ was used for identifying DE genes. Therefore, we can control the FDR at some pre-specified value (i.e. 0.05) by choosing the corresponding cutoff value $\hat {\gamma _{0}}$ as the minimum value of all cutoff values with an associated FDR no more than 0.05, or $\hat {\gamma }_{0}=\min \{\gamma _{0}: (\widehat {bFDR}(\gamma) \le 0.05)\}$.

## Results and discussion

In this section, two different simulation studies were conducted to investigate the performance of the proposed confident difference criterion methods on identifying DE genes for microarray or sequence-based studies, respectively. In addition, a real affymetrix dataset is used to further demonstrate the proposed methodology.

### Simulation study I: Microarray data

Two settings were considered. In the first setting, the intensity values having different means and variances between two biological conditions on each DE gene are simulated. In the second setting, the data were simulated from three biological conditions, with DE genes having different mean and variance values between at least two biological conditions.

#### Setting 1 (Two conditions)

Fifty simulations were used in this study to investigate the performance of different versions of the confident difference criterion methods described in the Confident difference criterion section. In each simulation, there were 5000 genes in total and 500 DE genes with 10 replications under each of the two biological groups. The log-scaled data were generated via $x_{g1k} \overset {\text {iid}}{\sim } \mathcal {N}(\mu _{g}-0.5 \delta _{g}, 0.2^{2}), x_{g2k} \overset {\text {iid}}{\sim } \mathcal {N}\left (\mu _{g}+0.5\delta _{g}, 0.9^{2}\right)$ with *δ*_*g*_=1,∀*g*=1,…,250 and *δ*_*g*_=−1,∀*g*=251,…,500 for the DE genes, and $x_{g1k},x_{g2k} \overset {\text {iid}}{\sim } \mathcal {N}(\mu _{g}, 0.7^{2})$ for the remaining EE genes. The average intensities *μ*_*g*_ were generated from an uniform distribution, where $\mu _{g} \overset {\text {iid}}{\sim } \mathcal {U} (5,11)$ for all genes. Conditionally conjugate priors described in the Model for microarray data subsection were used for all parameters *μ*_*g*_, *δ*_*g*_, $\sigma _{g1}^{2}$, $\sigma _{g2}^{2}$ and ${\sigma _{g}^{2}}$.

The simulated data were analyzed using both Methods I and II of the confident difference criteroin methods. For each version, the cutoff value *γ*_0_ were set to be 0.4, 0.6, or the cutoff value controlling the FDR to be no more than 0.05, separately. The genes with the calculated posterior distribution values *γ*_*g*_ via Equation (1) or Equation (3) less than the chosen *γ*_0_ were identified to be DE. To evaluate the performance of the confident difference criterion methods, the simulated datasets were also analyzed by four existing methods: Significant Analysis of Microarrays (SAM) [33], Linear Models for Microarray Data (LIMMA) [28], Semiparametric Hierarchical Method (SPH) [23], Empirical Bayesian Analysis of Gene Expression Data (EBarrays or EBA) [14]. All these existing methods allowed a control of the FDR for multiple comparisons. The genes were declared to be DE with FDR controlled at 0.05 for all these four methods.

Based on the identified gene list by each method, we calculated the number of claimed DE genes (CDE), the number of correctly claimed DE genes (CCDE), the number of correctly claimed EE genes (CCEE), the false positive rate (FPR), false negative rate (FNR), false discovery rate (FDR) and false non-discovery rate (FNDR) for all considered methods. These results and their standard deviations reported in parentheses were summarized in Table 1. Note, for Methods I and II, the choice of *γ*_0_=0.4 identified the a larger number of DE genes when compared to the choice of *γ*_0_=0.6. While for the case with FDR control of 0.05, the Method II identified the largest number of DE genes among all six methods. We also compared the results of the confident difference criterion method with a control of FDR against all four existing methods. We expected a method with good performance will provide a good control of FDR and provide smaller error rates in terms of FPR, FNR and FNDR. Under both versions of the confident difference criterion method, the achieved FDR is close to but less than 0.05, implying that the proposed confident difference criterion methods provided a good control of the FDR. All four existing methods also obtained a control of FDR at 0.05 successfully, although the SPH method provides a conservative control of FDR with the empirical FDR equal to 0.02. Since all methods provided small error rates of FPR and FNDR, we put more weight to the comparison of the empirical FNRs among all applied methods. The results in Table 1 showed that Method II provided the smallest empirical FNR out of all methods by successfully identifying almost all truly DE genes; and Method I had comparable empirical FNR as the SAM and the LIMMA methods, and much smaller empirical FNR when compared to the SPH and the EBA methods.

**Table 1:** Performance evaluation under Study I (Setting 1), (*G*=5000, 500 DE gene) ^*#*^

Cut-off	Method	CDE	CCDE	CCEE	FNR	FPR	FDR	FNDR
*γ*_0_	I	796.7(14.5)	466.7(4.7)	4169.9(13.9)	0.067(0.009)	0.073(0.003)	0.414(0.011)	0.008(0.001)
(0.4)	II	864.4(13.8)	499.2(1.1)	4134.8(13.8)	0.002(0.002)	0.081(0.003)	0.422(0.009)	0.000(0.000)
*γ*_0_	I	526.5(9.8)	419.2(6.7)	4392.7(6.9)	0.162(0.013)	0.024(0.002)	0.204(0.011)	0.018(0.001)
(0.6)	II	582.3(7.3)	493.3(3.0)	4411.0(6.9)	0.013(0.006)	0.020(0.002)	0.153(0.010)	0.002(0.001)
FDR	I	296.4(13.7)	283.3(12.9)	4486.9(2.9)	0.433(0.026)	0.003(0.001)	0.044(0.009)	0.046(0.003)
(0.05)	II	469.2(13.1)	450.7(10.2)	4481.5(4.4)	0.099(0.020)	0.004(0.001)	0.039(0.009)	0.011(0.002)
	SAM	330.5(16.0)	314.0(13.5)	4483.4(4.7)	0.372(0.027)	0.004(0.001)	0.050(0.013)	0.040(0.003)
	LIMMA	320.2(15.2)	304.9(13.7)	4484.7(4.1)	0.390(0.027)	0.003(0.001)	0.048(0.012)	0.042(0.003)
	SPH	192.0(10.6)	188.1(10.0)	4496.1(2.1)	0.624(0.020)	0.001(0.000)	0.020(0.011)	0.065(0.002)
	EBA	166.4(14.1)	158.8(13.3)	4492.3(2.2)	0.682(0.027)	0.002(0.000)	0.046(0.012)	0.071(0.003)

^#^Empirical estimates of the standard deviation were reported in the parentheses

#### Setting 2 (Three conditions)

The data were simulated from three biological conditions, and the first biological condition was considered as the reference group. A gene was set to be DE so that at least one group would be either up or down regulated from the reference group. Specifically, 500 DE genes out of 5000 genes were set in the data, and the log intensities of the DE genes were generated via $x_{g1k} \overset {\text {iid}}{\sim } \mathcal {N}\left (\mu _{g1}, 0.2^{2}\right), x_{g2k} \overset {\text {iid}}{\sim } \mathcal {N}\left (\mu _{g1}+0.5\nu _{g1}, 0.5^{2}\right), x_{g3k} \overset {\text {iid}}{\sim } \mathcal {N}\left (\mu _{g1}+0.5 \nu _{g2}, 0.8^{2}\right)$. Depending on whether the gene was set to be DE in one or both conditions from reference group, the parameters *ν*_*g*1_ and *ν*_*g*2_ were set to have *ν*_*g*1_=*ν*_*g*2_=1.5 for *g*=1,⋯,62 (up-regulated in both conditions); *ν*_*g*1_=1.5, *ν*_*g*2_=0 for *g*=63,⋯,125 (only up-regulated in condition 2); *ν*_*g*1_=1.5, *ν*_*g*2_=−1.5 for *g*=126,⋯,187 (up-regulated in condition 2, down-regulated in condition 3); *ν*_*g*1_=0 and *ν*_*g*2_=1.5 for *g*=188,⋯,250 (only up-regulated in condition 3); *ν*_*g*1_=0, *ν*_*g*2_=−1.5 for *g*=251,⋯,312 (only down-regulated in condition 3); *ν*_*g*1_=−1.5, *ν*_*g*2_=1.5 for *g*=313,⋯, 375 (down-regulated in condition 2, up-regulated in condition 3); *ν*_*g*1_=−1.5, *ν*_*g*2_=0 for *g*=376, ⋯,437 (only down-regulated in condition 2); *ν*_*g*1_=*ν*_*g*2_=−1.5, for *g*=438,⋯,500 (down-regulated in both conditions). The remaining genes were EE and their log intensities were generated via $x_{\textit {gtk}} \overset {\text {iid}}{\sim } \mathcal {N}\left (\mu _{g}, 0.6^{2}\right)$ for *t*=1,2,3 and *g*=501,⋯,5000. On all genes, the parameter *μ*_*g*1_ were generated from an uniform distribution, i.e., $\mu _{g1} \overset {\text {iid}}{\sim } \mathcal {U}(5,11)$. Each condition contained 10 replicates on each gene and 50 simulations were conducted.

The model similar to those described in Model for microarray data subsection and the proposed confident difference criterion methods including the Method I for comparing mean expressions and the Method II comparing both mean and variance expressions were applied to the simulated data. We considered three choices for the cutoff value *γ*_0_, including prespecified values 0.4, 0.6, or a value with FDR controlled at 0.05, separately. The data were also analyzed by the SAM [33], LIMMA [28], and EBArrays [14] with the FDR controlled at 0.05. The SPH [23] were not used in the study as they were proposed for studies with two biological conditions only. The analytical results from all methods were compared based on four error rates including FPR, FNR, FDR and FNDR from each considered method (Table 2). The confident difference criterion methods including Method I and Method II as well as the existing methods except LIMMA all provided an empirical FDR no more than 0.05 successfully. Comparing to the existing methods, the proposed confident difference criterion methods provided comparable FPR and smaller FNR and FNDR. Method II of the confident difference criterion method compares both mean and variance values of the gene expression intensities across different biological conditions. This is a potential reason for the proposed method providing smaller FNR for microarray data analysis. The confident difference criterion method is particularly effective when both mean and variance of the expression intensities differ across biological conditions on the DE genes.

**Table 2:** Performance evaluation under Study I (Setting 2), (*G*=5000, 500 DE gene) ^*#*^

Cut-off	Method	CDE	CCDE	CCEE	FNR	FPR	FDR	FNDR
*γ*_0_	I	1086.5(22.5)	476.3(4.4)	3890.8(21.9)	0.045(0.009)	0.135(0.005)	0.561(0.009)	0.006(0.001)
(0.4)	II	1388.1(25.7)	499.7(0.5)	3611.6(25.7)	0.001(0.001)	0.197(0.006)	0.640(0.007)	0.000(0.000)
*γ*_0_	I	656.8(13.8)	448.2(5.5)	4291.4(13.1)	0.104(0.011)	0.046(0.003)	0.317(0.014)	0.012(0.001)
(0.6)	II	749.3(15.0)	496.0(1.8)	4246.7(15.1)	0.008(0.004)	0.056(0.003)	0.338(0.014)	0.001(0.000)
FDR	I	357.1(8.7)	342.7(8.0)	4485.6(3.9)	0.315(0.016)	0.003(0.001)	0.040(0.011)	0.034(0.002)
(0.05)	II	480.7(10.5)	458.7(7.7)	4478.0(5.7)	0.083(0.015)	0.005(0.001)	0.046(0.011)	0.009(0.002)
	SAM	326.9(12.9)	312.0(11.2)	4485.1(4.5)	0.376(0.022)	0.003(0.001)	0.045(0.013)	0.040(0.002)
	LIMMA	329.5(51.9)	309.9(21.6)	4480.4(31.8)	0.380(0.043)	0.004(0.007)	0.053(0.045)	0.041(0.004)
	EBA	190.4(6.7)	184.2(6.7)	4493.7(1.9)	0.632(0.013)	0.001(0.000)	0.033(0.010)	0.066(0.001)

^#^Empirical estimates of the standard deviation were reported in the parentheses

### Simulation Study II: sequence-based data

The focus of this study is to investigate the performance of the proposed confident difference criterion method for identifying DE genes from sequence-based high-throughput experiments including SAGE and RNA-Seq studies.

#### Setting 1 (SAGE experiment)

The simulation proposed by Lu et al. [20] was used to conduct the simulation study. Specifically, 5000 genes were sampled under five libraries from each of the two conditions with fixed library sizes sampled uniformly between 30000 and 90000. A total of 500 genes were set to be DE genes. The data were generated from a negative binomial distribution, $y_{\textit {gtk}} \overset {\text {iid}}{\sim } \mathcal {NB}(\phi _{t}, \frac {m_{\textit {tk}}\lambda _{\textit {gt}}}{\phi _{t}+m_{\textit {tk}}\lambda _{\textit {gt}}})$ for gene *g*, for a fixed library *k* of condition *t*, where *m*_*tk*_ was the library size for library *k* under condition *t*; *ϕ*_1_ and *ϕ*_2_ denoted the dispersion parameters for data from the two conditions separately, and both set to be 0.4; *λ*_*gt*_ measured the expression level of gene *g* under condition *t* and were set with different values when gene *g* is DE and the same value when gene *g* is EE. For *g*=1,⋯,250, we set *λ*_*g*1_=8*E*−4 and *λ*_*g*2_=2*E*−4 to include down-regulated genes in condition 2. For *g*=251,⋯,500, we set *λ*_*g*1_=2*E*−4 and *λ*_*g*2_=8*E*−4 to include up-regulated genes in condition 2. For other genes with *g*=501,⋯,5000, we set *λ*_*g*1_=*λ*_*g*2_=2*E*−4 to include EE genes. Fifty simulations were used in this study.

The proposed confident difference criterion method for RNA-Seq data was used to analyze the simulated data. The posterior probability *γ*_*g*_ measuring the evidence of differential gene expression were estimated using average value of its posterior sampled values. The cutoff value *γ*_0_ for *γ*_*g*_ were set to be 0.4, 0.6 or a value to control the FDR to be 0.05, separately. The genes with estimated *γ*_*g*_ less than the chosen *γ*_0_ value were claimed to be DE. We also fit several other existing methods including edgeR [26], DESeq [2], BaySeq [10], NBPSeq [8], EBSeq [17], NOISeq [32], SAMSeq [19], and TSPM [3]. When the edgeR method was applied, we chose both options to estimate the common dispersion parameter for all tags and the tag-wise dispersion parameters respectively. For the NOISeq method, we estimated and controlled the FDR using the method proposed by Newton et al. [23] for identifying DE genes.

The results using the proposed confident difference criterion methods and all fitted existing methods for RNA-Seq data were summarized in Table 3. Similar to the simulation study I for microarray data, Table 3 showed that the higher the cutoff value *γ*_0_, the less number of genes were identified to be DE. The confident difference criterion method with a control of FDR at 0.05 achieved an empirical FDR of 0.044, and successfully identified 328.8 genes (65.8 %) on average out of 500 truly DE genes. Compared to other considered methods, the confident difference criterion method performed the best by providing the smallest FNR and FNDR while maintaining comparable FPR and a well controlled FDR. Out of the applied existing methods, the NOISeq method and edgeR method achieved the lowest FNR, and a FDR of no more than 0.05. The BaySeq method provided a conservative control of FDR, and achieved an empirical FDR of lower than 0.001 when controlling the FDR at 0.05. The DESeq, EBSeq and TSPM methods failed to control the FDR at 0.05. The SAMSeq method and TSPM method failed to identify most of the truly DE genes as DE genes, which was not surprising as the performance of both the SAMSeq and TSPM methods is highly sample size dependent as pointed out by Soneson and Delorenzi (2013) [29].

**Table 3:** Performance evaluation under Study II (Setting 1), (*G*=5000, 500 DE gene) ^*#*^

Method	CDE	CCDE	CCEE	FNR	FPR	FDR	FNDR
twocri. (*γ*_0_=0.4)	785.1(8.3)	465.6(2.5)	4180.5(7.6)	0.069(0.005)	0.071(0.002)	0.407(0.006)	0.008 (0.001)
(*γ*_0_=0.6)	509.4(6.8)	421.3(4.6)	4411.9(4.1)	0.157(0.009)	0.019(0.001)	0.173(0.007)	0.018(0.001)
(*ζ*=0.05)	344.2(8.2)	328.8(6.3)	4484.6(2.6)	0.342(0.013)	0.003(0.001)	0.044(0.007)	0.037(0.001)
edgeR ^1^(*ζ*=0.05)	289.7(17.6)	278.1(16.5)	4488.3(3.6)	0.444(0.033)	0.003(0.001)	0.040(0.011)	0.047(0.003)
edgeR ^2^(*ζ*=0.05)	290.6(18.1)	276.4(16.8)	4485.8(3.8)	0.447(0.034)	0.003(0.001)	0.049(0.012)	0.047(0.003)
DESeq(*ζ*=0.05)	297.2(21.3)	265.9(18.4)	4468.7(5.6)	0.468(0.037)	0.007(0.001)	0.105(0.016)	0.050(0.002)
BaySeq(*ζ*=0.05)	203.1(22.8)	203.0(22.8)	4499.9(0.2)	0.594(0.046)	0.000(0.000)	0.000(0.001)	0.062(0.004)
NBPSeq(*ζ*=0.05)	248.3(20.5)	239.8(19.3)	4491.5(4.0)	0.520(0.039)	0.002(0.001)	0.034(0.015)	0.055(0.004)
EBSeq(*ζ*=0.05)	303.7(18.8)	257.7(14.9)	4454.0(6.8)	0.485(0.030)	0.010(0.002)	0.151(0.017)	0.052(0.003)
NOISeq(*ζ*=0.05)	303.1(19.1)	294.4(17.6)	4491.3(3.2)	0.411(0.035)	0.002(0.001)	0.028(0.010)	0.044(0.004)
SAMSeq(*ζ*=0.05)	134.2(45.2)	126.1(43.2)	4491.9(3.4)	0.748(0.086)	0.002(0.001)	0.061(0.022)	0.077(0.008)
TSPM(*ζ*=0.05)	85.4 (19.2)	58.7 (15.4)	4473.3(6.5)	0.883(0.031)	0.006(0.001)	0.316(0.056)	0.090(0.003)

^#^Empirical estimates of the standard deviation were reported in the parentheses

edgeR ^1^estimates the common dispersion parameter for all tags; edgeR ^2^ estimates the tag-wise dispersion parameters

^*ζ*^denotes the FDR

#### Setting 2 (RNA-Seq experiment)

We used a similar simulation setting proposed by Kvam et al. [16] for illustrating the application of the proposed confident difference criterion method for RNA-Seq experiment. We still simulated 50 dataset, each dataset contained six libraries with three libraries from each of the two conditions on 5000 genes, among which 250 genes were set to be up-regulated genes and another 250 genes were set to be down-regulated genes in condition 2 versus condition 1. The overall mean expression levels across both conditions were generated from a gamma distribution with $\lambda _{g} \sim \mathcal {G}(0.25, 600)$. To avoid including genes with low expression levels from both conditions as DE genes, we set the difference in the gene expression levels between conditions in two ways depending on whether the value of *λ*_*g*_ is larger than one. Specifically, we generated *ξ*_*g*_ from uniform distribution $\mathcal {U}(3,20)$ for each gene. If the value of *λ*_*g*_>1, we let the fold change between the gene expression values of DE genes to be *ξ*_*g*_, or $\lambda _{g1}=\lambda _{g}/\sqrt {\xi _{g}}$ and $\lambda _{g2}=\lambda _{g}*\sqrt {\xi _{g}}$ for up-regulated genes, and $\lambda _{g1}=\lambda _{g}*\sqrt {\xi _{g}}$ and $\lambda _{g2}=\lambda _{g}/\sqrt {\xi _{g}}$ for down-regulated genes. If the value of *λ*_*g*_≤1, we let the absolute difference in the gene expression values to be *ξ*_*g*_, or we let *λ*_*g*1_=*λ*_*g*_+*ξ*_*g*_ and *λ*_*g*2_=*λ*_*g*_ for down-regulated genes, and *λ*_*g*1_=*λ*_*g*_ and *λ*_*g*2_=*λ*_*g*_+*ξ*_*g*_ for up-regulated genes in condition 2. For an EE gene, we had *λ*_*g*1_=*λ*_*g*2_=*λ*_*g*_.

Then we generated the data using negative binomial distribution of $y_{\textit {gtk}} \overset {\text {iid}}{\sim } \mathcal {NB}(\phi _{t}, \frac {\lambda _{\textit {gt}}}{\phi _{t}+\lambda _{\textit {gt}}})$ for gene *g*, and the overdispersion parameters *ϕ*_1_ and *ϕ*_2_ were set to have *ϕ*_1_ = 1 and *ϕ*_2_ = 8 respectively for DE genes; and *ϕ*_1_=*ϕ*_2_=4 for EE genes.

All methods applied in setting I of simulation study II were also used for data analysis in this simulation study. The results in Table 4 displayed that the confident difference criterion method with a control of FDR at 0.05, the edgeR method with common dispersion parameter over genes, the edgeR with gene-wise dispersion parameter, the BaySeq, the NBPSeq, the NOISeq methods successfully controlled the FDR at 0.05. Additionally the confident difference criterion method, the NBPSeq method, the edgeR method with a common dispersion parameter over genes also provided a good and comparable control of FNR of less than 0.2, while maintaining low levels of FPR and FNDR.

**Table 4:** Performance evaluation under Study II (Setting II), (*G*=5000, 500 DE gene) ^*#*^

Method	CDE	CCDE	CCEE	FNR	FPR	FDR	FNDR
twocri.(*γ*_0_=0.4)	654.6(5.4)	460.9(2.3)	4306.3(5.2)	0.078(0.005)	0.043(0.001)	0.295(0.006)	0.009(0.000)
(*γ*_0_=0.6)	490.0(3.9)	434.1(2.4)	4444.2(3.4)	0.132(0.005)	0.012(0.001)	0.114(0.006)	0.014(0.001)
(*ζ*=0.05)	415.7(5.0)	400.6(3.5)	4484.9(2.7)	0.199(0.007)	0.003(0.001)	0.036(0.006)	0.022(0.001)
edgeR ^1^(*ζ*=0.05)	420.0(8.6)	411.7(8.4)	4491.7(2.8)	0.177(0.017)	0.002(0.001)	0.020(0.001)	0.020(0.002)
edgeR ^2^(*ζ*=0.05)	399.4(10.2)	386.3(9.8)	4486.9(4.6)	0.227(0.020)	0.003(0.001)	0.033(0.011)	0.025(0.002)
DESeq(*ζ*=0.05)	443.6(15.9)	409.3(15.1)	4465.8(5.3)	0.181(0.030)	0.008(0.001)	0.077(0.011)	0.020(0.003)
BaySeq(*ζ*=0.05)	331.0(15.5)	327.0(14.9)	4496.0(2.2)	0.346(0.030)	0.001(0.000)	0.012(0.006)	0.037(0.003)
NBPSeq(*ζ*=0.05)	422.3(7.9)	412.7(7.9)	4490.4(3.1)	0.175(0.016)	0.002(0.001)	0.023(0.007)	0.019(0.002)
EBSeq(*ζ*=0.05)	332.9(14.0)	248.4(11.1)	4415.6(9.4)	0.503(0.022)	0.019(0.002)	0.253(0.023)	0.054(0.002)
NOISeq(*ζ*=0.05)	196.8(11.0)	191.4(10.8)	4494.6(2.5)	0.617(0.022)	0.001(0.001)	0.028(0.013)	0.064(0.002)
SAMSeq(*ζ*=0.05)	274.3(15.7)	212.1(7.7)	4437.8(10.9)	0.576(0.015)	0.014(0.002)	0.226(0.029)	0.061(0.001)
TSPM(*ζ*=0.05)	129.9(10.5)	80.1 (8.9)	4450.2(7.1)	0.840(0.018)	0.011(0.002)	0.383(0.046)	0.086(0.002)

^#^Empirical estimates of the standard deviation were reported in the parentheses

edgeR ^1^ estimates common dispersion parameter for all tags; edgeR ^2^ estimats tag-wise dispersion parameters

^*ζ*^denotes the false discovery rate

### Real data analysis

We used a real data set obtained using customized Bovine Affymetrix arrays (Davis, Talbott, Yu, and Cupp, unpublished results) to illustrate the proposed method. Fifteen arrays composed of three replicate arrays under three biological conditions were produced to screen for DE genes associated with prostaglandin *F*2*α*(PGF) treatment after 30 min, 1 h, 2 h, and 4 h compared to the control treatment (saline). For simplicity, we focused on detecting genes using the confident difference criterion methods (Method I and Method II) that were regulated 1 h or 2 h after PGF treatment. The data were extracted, normalized and summarized using the Robust Multi-array Average (RMA) [12] method at the exon level via the Affymetrix expression console. The data set contains 21724 genes. Note that some genes may have multiple probe replicates ranging from one replicate to 266 replicates, and the data from different probes of the same gene may have large variation even after RMA normalization. We centered the data from each probe of the same gene to the mean log intensities of that gene, and excluded 3116 genes with only a single probe replicate from the analysis to make sure that the parameters were estimable. Additionally, we excluded 2137 low expression genes if two-thirds or more (six out of nine) samples on this gene had gene expression values measured by the geometric mean expression values across different probes less than 10. Of the remaining 16471 genes with replicate probes, we used *z*_*gjtk*_ to denote the *k* ^*th*^ biological replicate sample of the log2 scale gene expression intensity for probe *j* of gene *g* under condition *t*. Note that the index *j* was added to the previous notations for the log intensity values as data are available for multiple probes on the same gene. We assumed normal distribution for the log2 intensities with $z_{\textit {gjtk}} \sim \mathcal {N}(\mu _{\textit {gtk}}, \sigma ^{2\ast }_{\textit {gt}})$, and the same prior for *μ*_*gtk*_ as what we set for ***X***_*gt*_ in the Model for microarray data subsection. The variance parameters are assumed to follow inverse gamma distribution with $\sigma ^{2\ast }_{\textit {gt}} \sim \mathcal {IG}(\alpha ^{\ast }_{t},\beta ^{\ast }_{t})$ with $\alpha ^{\ast }_{t}=2$ and $\beta ^{\ast }_{t} \sim \mathcal {G}(\alpha ^{\ast }_{0},\beta ^{\ast }_{0})$. We set $\alpha ^{\ast }_{0}=1$ and $\beta ^{\ast }_{0}\sim IG(\alpha ^{\ast },\beta ^{\ast })$ where both *α* ^∗^=*β* ^∗^=1. During computation for controlling the FDR, we reuse these settings of the prior distributions on the parameters *μ*_*gtk*_ and $\sigma ^{2\ast }_{\textit {gt}}$ for DE genes. For EE gens, we assume that $z_{\textit {gjtk}} \sim \mathcal {N}(\mu _{\textit {gtk}},\sigma ^{2\ast }_{g})$, and make similar augment for the prior distributions of their parameters *μ*_*gtk*_ and $\sigma ^{2\ast }_{g}$ as the DE genes. The proposed confident difference criterion methods were applied to assess the evidence of differential expression on each gene and identify DE genes with the cutoff value equal to be 0.4, 0.6 or a value that controls the FDR at 0.05.

**Fig. 2:**
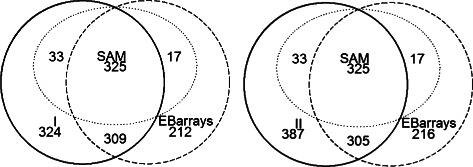
Number of identified DE genes out of 16471 genes from real data analysis. Two venndiagrams present the overlapping among the DE genes identified separately by Method I/II, SAM, and EBarrays with the false discovery rate controlled at 0.05 from the real data

In addition, we analyzed the real data using the existing methods including SAM, LIMMA, and EBarrays as described in the Simulation Studies section for identification of DE genes. Since the existing methods were developed for data with single probe replicate on each gene, we calculated the mean log intensities over all probes for each biological sample on each gene to quantify the corresponding gene expression. The genes were declared to be DE if the false discovery rate was no more than 0.05. We used Venn diagrams to demonstrate the overlap of DE genes identified by Method I (Fig. 2, Left Panel) or Method II (Fig. 2, Right Panel), to the DE genes identified by SAM and EBarrays (Fig. 2). The results showed that more genes were identified to be DE by the proposed Method I and Method II than the existing methods. Specifically, 1050 DE genes were identified by Method II, while 896 genes were identified to be DE by either SAM or EBarrays. Of note 340 out of 353 DE genes identified by LIMMA were also identified by SAM (data not shown), and 951 of 991 DE genes identified by Method I were also identified as DE by Method II. We found that SAM identified 375 DE genes, all of which were also identified by other methods. For example, 358 (95.5 %) genes identified by SAM were also identified by Method I or II; and 342 (91.2 %) genes identified by SAM were also identified by EBarrays method. The EBarrays method identified 863 DE genes, out of which, 643 (74.5 %) genes were also identified by Method I or II. Method I identified 116 of the 324 genes identified by LIMMA when comparing all four time points versus control in the same dataset, while Method II called 105 out of 387 genes DE that were also called DE by LIMMA within the whole dataset. In addition, many genes identified to be DE only by Method II not by Method I show a linear trend among the average gene expression across conditions observed from samples collected with longer time after treatment, and larger data variations under the control condition than those observed at other time points after treatment. For example, the average log2 gene expression of THBS1 increased from 9.22 under control condition to 10.35 at 2h after treatment, and the standard deviation equaled 0.88 under the control condition, and 0.37 at 2 h after treatment. This gene was only detected to be DE by Method II and was shown to play roles in angiogenesis [37].

The genes identified solely by Method I or Method II were analyzed by Ingenuity Pathway Analysis (IPA, Build version: 313398M, Content version: 18841524 (Release Date: 2014-06-24) to determine biological functions and pathways associated with the newly identified genes. Genes identified solely by Method I and not by SAM or EBarrays were analyzed by IPA which identified several major canonical pathways such as hepatic fibrosis / hepatic stellate cell activation, glucocortiocoid receptor signaling, agranulocyte adhesion and diapedesis, and role of IL-17A in arthritis (Additional file 2: Table S1). Many of the canonical pathways identified have either established or potential roles in corpus luteum function indicating that Method I identified DE genes that are biologically relevant within the model. Method I also identified IL1B (*P*=2.12*E*−08) and TNF (*P*=3.03*E*−08) as upstream regulators of the genes found exclusively by Method I, which also fits with known and suspected mechanisms of PGF action within the corpus luteum [1, 24].

Genes identified solely by Method II were also analyzed by IPA which identified canonical pathways such as hepatic fibrosis/hepatic stellate cell activation [21], glucocorticoid receptor signaling, IL-8 signaling, and granulocyte adhesion and diapedesis. Upstream regulators of gene found solely by Method II included: IL1B (*P*=4.56*E*−13), TGFB1 (*P*=1.19*E*−11), and IFNG (*P*=1.82*E*−11). The IPA results both concur with current literature and offer new insights into the possible mechanism(s) of action of PGF in the corpus luteum [1, 9, 11, 21]. These and similar canonical and regulatory functions were also identified when the complete dataset (30 min, 1 h, 2 h, and 4 h) was analyzed by IPA. These network functions are in agreement with the known or suspected changes in biological function in the corpus luteum following PGF treatment in several species [1, 5, 22, 27]. Several of the genes identified by Methods I and II are known to be involved in regulation of the fate of the corpus luteum after PGF treatment, and were also identified as DE genes in our larger data set and a similar microarray dataset examining the effects of PGF in the cow [22]. For example, genes that code for chemokines (e.g., CCL3 and CCL8) and prostaglandin synthesis (e.g., PTGS2) were found to be significantly up-regulated at 1 and 2 h using Methods I and II which were not identified using LIMMA. However, CCL3, CCL8, and PTGS2 were all identified as significantly up-regulated in later time points using LIMMA, which conservatively identifies DE genes. Therefore, it seems possible that Methods I and II may provide a more sensitive approach to identify the temporal patterns of gene expression.

## Conclusion

In this paper, we have proposed a new differentially expressed gene selection algorithm, which controls the FDR based on predictive Bayesian estimates. The simulation studies empirically showed that the proposed confident difference criterion methods outperform the existing methods when comparing gene expressions across different conditions for both microarray studies and sequence-based high-throughput studies. For the analysis of the real data, the method II successfully identified more clinically important DE genes than the other methods. In comparison to Method I, the Method II provides a much better sensitivity rate, but slightly a lower specificity rate based on the simulation studies.

In scenarios where the data are not symmetrically distributed, we need to model the data with other types of distributions (e.g., a gamma distribution). The confident difference criterion method proposed for comparing both means and variances can also be extended to evaluate the differences in multiple parameters defined in the non-normal data distributions. In addition, the Euclidean distances used in the proposed confident difference criterion method may also be extended to other types of distances to measure the difference among the distributions under two or more biological conditions. In the case of two conditions, the entropy-based distance such as the Kullback-Leibler (KL) divergence may be considered. However, the distribution of the entropy-based statistics is quite difficult to characterize and, hence, it is quite challenging to choose the cutoff value for the entropy statistics. Such extensions need to be further investigated. Finally, we note that all models considered in this paper assume that the gene expressions are independent across genes. The proposed confident difference criterion methods do not require the independence assumption. However, the performance of the confident difference criterion methods under the correlated models need to be further examined.

## Availability and requirements

All analyses results presented in this paper were obtained using codes developed in FORTRAN with IMSL library. We have also implemented the proposed method in R for windows (32 bits). The R codes can be obtained at the websites: http://www.unmc.edu/publichealth/departments/biostatistics/facultyandstaff/cdc_micro.zipand http://www.unmc.edu/publichealth/departments/biostatistics/facultyandstaff/cdc_RNASeq.zip.

## Additional files

Additional file 1
**Methods.** Mathematical Proof for Propositions 1 and 2.

Additional file 2
**Real Data Analysis Results.** Canonical Pathways identified by Methods I and II.
