# Supplementary material for: Confident difference criterion: a new Bayesian differentially expressed gene selection algorithm with applications
Source: BMC Bioinformatics. 2015 Aug 7;16:245. doi: 10.1186/s12859-015-0664-3 (PMC4527130; doi:10.1186/s12859-015-0664-3)
Supplement: Additional file 1 — Methods. Mathematical Proof for Propositions 1 and 2. [file 12859_2015_664_MOESM1_ESM.pdf]

Additional File 1: Mathematical Proof for Propositions 1 and 2

Proof for Proposition 1

Let $N(\xi_{\mu_{g2}-\mu_{g1}}, \sigma^2_{\mu_{g2}-\mu_{g1}})$ denote the posterior distribution of $\mu_{g2}-\mu_{g1}$. Then we have

$$\gamma_g = Pr(\frac{|\mu_{g2}-\mu_{g1}|}{\sigma_{\mu_{g2}-\mu_{g1}}} > 2|D_{obs}) > \gamma_0 \Leftrightarrow Pr(-2 < \frac{\mu_{g2}-\mu_{g1}}{\sigma_{\mu_{g2}-\mu_{g1}}} < 2|D_{obs}) \leq 1-\gamma_0$$
$$\Leftrightarrow Pr(-2 - \frac{\xi_{\mu_{g2}-\mu_{g1}}}{\sigma_{\mu_{g2}-\mu_{g1}}} < \frac{\mu_{g2}-\mu_{g1}-\xi_{\mu_{g2}-\mu_{g1}}}{\sigma_{\mu_{g2}-\mu_{g1}}} < 2 - \frac{\xi_{\mu_{g2}-\mu_{g1}}}{\sigma_{\mu_{g2}-\mu_{g1}}}|D_{obs}) \leq 1-\gamma_0$$
$$\Leftrightarrow \Phi\Big(2 - \frac{\xi_{\mu_{g2}-\mu_{g1}}}{\sigma_{\mu_{g2}-\mu_{g1}}}\Big) - \Phi\Big(-2 - \frac{\xi_{\mu_{g2}-\mu_{g1}}}{\sigma_{\mu_{g2}-\mu_{g1}}}\Big) \leq 1-\gamma_0. \tag{0.1}$$

When $\xi_{\mu_{g2}-\mu_{g1}} \geq 0$, $\Phi\Big(-2 - \frac{\xi_{\mu_{g2}-\mu_{g1}}}{\sigma_{\mu_{g2}-\mu_{g1}}}\Big) < \Phi(-2)$ and using (0.1), we obtain $\Phi\Big(2 - \frac{\xi_{\mu_{g2}-\mu_{g1}}}{\sigma_{\mu_{g2}-\mu_{g1}}}\Big) \leq 1-\gamma_0+\Phi(-2)$, which implies $\frac{\xi_{\mu_{g2}-\mu_{g1}}}{\sigma_{\mu_{g2}-\mu_{g1}}} > 2-\Phi^{-1}(1+\Phi(-2)-\gamma_0)$. Thus, $\max\{Pr(\mu_{g2}-\mu_{g1} > 0\ |D_{obs}),\ Pr(\mu_{g2}-\mu_{g1} < 0\ |D_{obs})\} = \max\Big\{\Phi\Big(\frac{\xi_{\mu_{g2}-\mu_{g1}}}{\sigma_{\mu_{g2}-\mu_{g1}}}\Big), 1-\Phi\Big(\frac{\xi_{\mu_{g2}-\mu_{g1}}}{\sigma_{\mu_{g2}-\mu_{g1}}}\Big)\Big\} = \Phi\Big(\frac{\xi_{\mu_{g2}-\mu_{g1}}}{\sigma_{\mu_{g2}-\mu_{g1}}}\Big) > \Phi(2-\Phi^{-1}(1+\Phi(-2)-\gamma_0))$. When $\xi_{\mu_{g2}-\mu_{g1}} < 0$, we have $\Phi\Big(-2 + \frac{\xi_{\mu_{g2}-\mu_{g1}}}{\sigma_{\mu_{g2}-\mu_{g1}}}\Big) < \Phi(-2)$. It is easy to show that (0.1) can be rewritten as

$$\Phi\Big(2 + \frac{\xi_{\mu_{g2}-\mu_{g1}}}{\sigma_{\mu_{g2}-\mu_{g1}}}\Big) - \Phi\Big(-2 + \frac{\xi_{\mu_{g2}-\mu_{g1}}}{\sigma_{\mu_{g2}-\mu_{g1}}}\Big) \leq 1-\gamma_0. \tag{0.2}$$

Using (0.2), we obtain $\Phi\Big(2 + \frac{\xi_{\mu_{g2}-\mu_{g1}}}{\sigma_{\mu_{g2}-\mu_{g1}}}\Big) \leq 1+\Phi(-2)-\gamma_0$, which implies $-\frac{\xi_{\mu_{g2}-\mu_{g1}}}{\sigma_{\mu_{g2}-\mu_{g1}}} \geq 2-\Phi^{-1}(1+\Phi(-2)-\gamma_0)$. Thus, $\max\{Pr(\mu_{g2}-\mu_{g1} > 0\ |D_{obs}),\ Pr(\mu_{g2}-\mu_{g1} < 0\ |D_{obs})\} = 1-\Phi\Big(\frac{\xi_{\mu_{g2}-\mu_{g1}}}{\sigma_{\mu_{g2}-\mu_{g1}}}\Big) = \Phi\Big(-\frac{\xi_{\mu_{g2}-\mu_{g1}}}{\sigma_{\mu_{g2}-\mu_{g1}}}\Big) \geq \Phi\Big(2-\Phi^{-1}(1+\Phi(-2)-\gamma_0)\Big)$ when $\xi_{\mu_{g2}-\mu_{g1}} < 0$, which completes the proof.

Proof for Proposition 2

Let $d_g = \bar{x}_{g2} - \bar{x}_{g1}$. The conditional posterior distribution of the difference in the mean parameters of the intensities from the two biological conditions $\mu_{g2}-\mu_{g1}$ under the alternative hypothesis follow a normal distribution of $\delta_g \sim \mathcal{N}(U_{\delta_g} \times S^2_{\delta_g}, S^2_{\delta_g})$, where $U_{\delta_g} = \frac{n_g}{2\sigma_g^2} d_g$ and $S^{-2}_{\delta_g} = \frac{1}{\omega^2} + \frac{n_g}{2\sigma_g^2}$. The confident difference criterion method declares a gene to be DE if

$$\gamma_g = Pr\left(\frac{|\mu_{g2}-\mu_{g1}|}{\sigma_{\mu_{g2}-\mu_{g1}}} > 2\Big|D_{obs}\right) \geq \gamma_0. \tag{0.3}$$

After some algebra, we can show that (0.3) is equivalent to

$$|U_{\delta_g} S_{\delta_g}| = \Big|\frac{n_g}{2\sigma_g^2}(\bar{x}_{g1} - \bar{x}_{g2})\Big|\Big[\frac{1}{\omega^2} + \frac{n_g}{2\sigma_g^2}\Big]^{-\frac{1}{2}} = |d_g|\Big[\frac{2\sigma_g^2}{n_g}(\frac{2\sigma_g^2}{n_g\omega^2} + 1)\Big]^{-\frac{1}{2}} \geq |E_g^*|, \quad (0.4)$$

where the value of $|E_g^*|$ depends on the cut-off value γ_0 through $\Phi(2 + E_g^*) - \Phi(-2 + E_g^*) = 1 - \gamma_0$. Following Yu et al.[1], the CBF method declares a gene to be DE if the Bayes factor $BF_{01}(g) = \Big(\frac{n_g\omega^2}{2\sigma_g^2} + 1\Big)^{\frac{1}{2}} \exp\Big\{ - \frac{n_g d_g^2}{4\sigma_g^2\big(1 + \frac{2\sigma^2}{n_g\omega^2}\big)}\Big\} \leq C_0$, which is equivalent to

$$|d_g| \geq \sqrt{\frac{2\sigma_g^2}{n_g}\Big(1 + \frac{2\sigma^2}{n_g\omega^2}\Big)\Big[\log\big(\frac{n_g\omega^2}{2\sigma_g^2} + 1\big) - 2\log(C_0)\Big]}. \quad (0.5)$$

By choosing C_0 to satisfy

$$\Phi\Big(2 + \log\big(\frac{n_g\omega^2}{2\sigma_g^2} + 1\big) - 2\log(C_0)\Big) - \Phi\Big(- 2 + \log\big(\frac{n_g\omega^2}{2\sigma_g^2} + 1\big) - 2\log(C_0)\Big) = 1 - \gamma_0,$$

(0.4) and (0.5) are equivalent when $\log\big(\frac{n_g\omega^2}{2\sigma_g^2} + 1\big) > 2\log(C_0)$. Thus, the confident difference criterion method agrees with the CBF method. This completes the proof.

References

[1] Yu F, Chen M-H, Kuo L: Detecting differentially expressed genes using calibrated Bayes factors. Statistica Sinica 2008; 18: 783-802.
