# Supplementary material for: Confident difference criterion: a new Bayesian differentially expressed gene selection algorithm with applications
Source: BMC Bioinformatics. 2015 Aug 7;16:245. doi: 10.1186/s12859-015-0664-3 (PMC4527130; doi:10.1186/s12859-015-0664-3)
Supplement: Additional file 2 — Real Data Analysis Results. Canonical Pathways identified by Methods I and II. [file 12859_2015_664_MOESM2_ESM.pdf]

Supplemental Table 1: Canonical Pathways Identified by Methods I & II

	Name	p-value	Relevant Articles
Method I	Hepatic Fibrosis/Hepatic Stellate Cell Activation	5.31E-05	Maroni and Davis 2012 [7]
	Glucocorticoid Receptor Signaling	4.81E-04	Komiyama et al. 2008; Shenavai et al. 2012 [5, 12]
	MIF-mediated Glucocorticoid Regulation	7.88E-04	Gonzalez et al. 2010; Li et al. 2011 [3, 6]
	Agranulocyte Adhesion and Diapedesis	1.22E-03	Poole et al. 2012; Poole et al. 2013 [10, 11]
	Role of IL-17A in Arthritis	2.01E-03	Ozkan et al. 2014 [9]
Method II	Hepatic Fibrosis/Hepatic Stellate Cell Activation	1.83E-07	Maroni and Davis 2012 [7]
	Glucocorticoid Receptor Signaling	3.53E-04	Komiyama et al. 2008; Shenavai et al. 2012 [5, 12]
	IL-8 Signaling	5.09E-04	Atli et al. 2012; Talbott et al. 2014 [1, 14]
	Granulocyte Adhesion and Diapedesis	6.78E-04	Jiemtaweeboon et al. 2011; Shirasuna et al. 2012; Talbott et al. 2014 [4, 13, 14]
	Bladder Cancer Signaling	8.23E-04	Berisha et al., 2006; Neuvians et al., 2004 [2, 8]

References

[1] Atli MO, Bender RW, Mehta V, Bastos MR, Luo W, Vezina CM, Wiltbank MC: Patterns of gene expression in the bovine corpus luteum following repeated intrauterine infusions of low doses of prostaglandin $F_2\alpha$. Biol Reprod 2012; 86(4):130.

[2] Berisha B, Welter H, Shimizu T, Miyamoto A, Meyer HH, Schams D: Expression of fibroblast growth factor 1 (FGF1) and FGF7 in mature follicles during the periovulatory period after GnRH in the cow. J Reprod Dev 2006; 52(2):307-313.

[3] González F, Rote NS, Minium J, Weaver AL, Kirwan JP: Elevated circulating levels of macrophage migration inhibitory factor in polycystic ovary syndrome. Cytokine 2010; 51(3):240-244.

[4] Jiemtaweeboon S, Shirasuna K, Nitta A, Kobayashi A, Schuberth HJ, Shimizu T, Miyamoto A: Evidence that polymorphonuclear neutrophils infiltrate into the developing corpus luteum and promote angiogenesis with interleukin-8 in the cow. Reprod Biol Endocrin 2011; 9:79.

[5] Komiyama J, Nishimura R, Lee H-Y, Sakumoto R, Tetsuka M, Acosta TJ, Skarzynski DJ, Okuda K: Cortisol Is a Suppressor of Apoptosis in Bovine Corpus Luteum. Biol Reprod 2008; 78(5):888-895.

[6] Li C, Qiao B, Zhan Y, Qi W, Chen ZJ: First evidence of genetic association between the MIF-173G/C single-nucleotide polymorphisms and polycystic ovary syndrome. Am J Reprod Immunol 2011; 66(5):416-422.

[7] Maroni D, Davis JS: TGFB1 disrupts the angiogenic potential of microvascular endothelial cells of the corpus luteum. J Cell Sci 2012; 124(14):2501-2510.

[8] Neuvians TP, Berisha B, Schams D: Vascular endothelial growth factor (VEGF) and fibroblast growth factor (FGF) expression during induced luteolysis in the bovine corpus luteum. Mol Reprod Dev 2004; 67(4):389-395.

[9] Ozkan ZS, Deveci D, Kumbak B, Simsek M, Ilhan F, Sekercioglu S, Sapmaz E: What is the impact of Th1/Th2 ratio, SOCS3, IL17, and IL35 levels in unexplained infertility? J Reprod Immunol 2014; 103:53-58.

[10] Poole DH, Pate JL: Luteal microenvironment directs resident T lymphocyte function in cows. Biol Reprod 2012; 86(2):29.

[11] Poole DH, Ndiaye K, Pate JL: Expression and regulation of secreted phosphoprotein 1 in the bovine corpus luteum and effects on T lymphocyte chemotaxis. Reproduction 2013; 146(6):527-537.

[12] Shenavai S, Preissing S, Hoffmann B, Dilly M, Pfarrer C, Ȯzalp GR, Caliskan C, Seyrek-Intas K, Schuler G: Investigations into the mechanisms controlling parturition in cattle. Reproduction 2012; 144(2):279-292.

[13] Shirasuna K, Jiemtaweeboon S, Raddatz S, Nitta A, Schuberth HJ, Bollwein H, Shimizu T, Miyamoto A: Rapid accumulation of polymorphonuclear neutrophils in the Corpus luteum during prostaglandin F(2α)-induced luteolysis in the cow. PLoS One 2012; 7(1):e29054.

[14] Talbott H, Delaney A, Zhang P, Yu Y, Cushman RA, Cupp AS, Hou X, Davis JS: Effects of IL8 and immune cells on the regulation of luteal progesterone secretion. Reproduction 2014; 148(1):21-31.
